# The psychic envelopes in psychoanalytic theories of infancy

**DOI:** 10.3389/fpsyg.2014.00734

**Published:** 2014-07-15

**Authors:** Denis Mellier

**Affiliations:** Laboratoire de Psychologie, Département de Psychologie, UFR Sciences du Langage, de l'Homme et de la Société, Université de Franche-ComtéBesançon, France

**Keywords:** body image, infant development, intersubjectivity, psychoanalysis, non-verbal communication, self concept, Skin-Ego, containing function

## Abstract

This paper aims to review the topic of psychic envelopes and to sketch the main outlines of this concept in infancy. We first explore the origins of the concept in Freud's “protective shield” and then its development in adult psychoanalysis before going on to see how this fits in infancy with post-Bionian psychoanalysis and development. Four central notions guide this review: (1) Freud's “protective shield” describes a barrier to protect the psychic apparatus against potentially overflowing trauma. It is a core notion which highlights a serious clinical challenge for patients for whom the shield is damaged or faulty: the risk of confusion of borders between the internal/external world, conscious/unconscious, mind/body, or self-conservation/sexuality. (2) Anzieu's “Skin-Ego” is defined by the different senses of the body. The different layers of experienced sensation, of this body-ego, go on to form the psychic envelope. This theory contributes to our understanding of how early trauma, due to the failures of maternal care, can continue to have an impact in adult life. (3) Bick's “psychic skin” establishes the concept in relation to infancy. The mother's containing functions allow a first psychic skin to develop, which then defines an infant's psychic space and affords the infant a degree of self-containment. Houzel then conceptualized this process as a stabilization of drive forces. (4) Stern's “narrative envelope” derives from the intersection between psychoanalysis and neuroscience. It gives us another way to conceptualize the development of pre-verbal communication. It may also pave the way for a finer distinction of different types of envelopes. Ultimately, in this review we find that psychic envelopes in infancy can be viewed from four different perspectives (economic, topographical, dynamic, and genetic) and recommend further investigation.

Although it has received relatively little attention elsewhere, the concept of psychic envelopes is being used in psychoanalysis in France. The aim of the present article is to undertake a broad investigative review of this concept, and to specify its main characteristics, particularly as applied in understanding infant development. To facilitate this we will focus on four notions in which the concept is rooted: the protective shield (Freud), the Skin-Ego (Anzieu), the psychic skin (Bick) and the narrative envelope (Stern).

## The “protective shield” (freud) and trauma

After considering the working of a “contact barrier” in his (1894) model of the psyche, Freud (1950/1920) introduced the idea of a protective function and filter against excitations from the external world. This “protective shield” operated to protect the mental apparatus from external excitations that could overwhelm it, and so kept the capacity of attention toward the external world intact. Freud used the metaphor of the external layer of a primitive organism (an “undifferentiated vesicle of sensitive substance”), which is sacrificed on impact. Having first (1894) considered trauma as having a sexual origin, Freud (1950/1894) emphasized the infant's need for care. After the First World War he again revised his theory of anxiety. In 1926 he described the initial infantile situation as one of “helplessness” (*Hilflogsigkeit*), where this can be seen as a sort of generalization of the early risk of trauma (Freud, 1950/1926). If an infant needed protection, help, or attention, but lacked a “protective shield,” he would be overwhelmed. This notion of a protective shield is at the core of the notion of a psychic envelope.

## Freud's successors and the limits of mental functioning

Among four of those who followed Freud—Winnicott, Bion, Marty and Laplanche—we find four different ways to understand the notions of helplessness and a protective shield or psychic envelope that keeps the infantile mental apparatus from being overwhelmed. Each highlighted the role of the mother, but they characterized the notion of overwhelming differently. I think that each author could have spoken of a psychic envelope, but in fact this specific expression was rarely used.

Winnicott (1958, 2005) related psychic conflict to the border of the psyche, between “inside” and “outside,” in what he described as an “intermediate zone,” a transitional space. Only a maternal object that is “good enough” can provide containment for the baby's psyche, which is torn between the “needs of the ego” and the sexual drives, in order to maintain a “continuity of being.” The baby develops by becoming the center of gravity of maternal care, in which a “border membrane” is established between the “inside” and the “outside.”

According to Winnicott, what we are here calling the “envelope” or “container” is situated at the level of the self, in relation to challenges of satisfaction of the drives. In his case “overwhelming” results from an impingement of the instinct upon the self and the needs of ego. The risk is confusion: between narcissism and sexuality; between the self and the environment; and between the inner world and the external world. If the infant's defenses are overwhelmed the result is a false organization of the self, called “the false self.” Premature and intrusive satisfactions on the part of the baby nourish this “false self,” with the aim of protecting the secret and real self. This development precludes a “good enough” differentiation between the self and the environment.

In the context of therapy, Winnicott describes the difference between neurotics and psychotics as holding between those who had the benefit of a “good enough mother” and those who had suffered from impingement. In the latter case, just as the baby needed to experience good enough caregivers, so the patient needed to experience a “good enough” therapeutic arrangement. So he writes that if the couch *represents* a symbol of maternal love for the neurotic, it is *experienced* physically as loving and caring by the psychotic (Winnicott, 1953). The baby needs to experience good enough caregivers; the construction of its self depends on that process.

Bion (1962, 1970) developed a new paradigm in psychoanalysis in terms of the containing function, which we can here also describe as that of a psychic envelope. He introduced the idea of a “membrane” in the context of treating psychosis (Bion, 1962). In psychoanalytic therapy psychotic individuals show a double confusion: in the delimitation between the conscious and the unconscious and in that between the patient and the analyst. In therapy the analyst must establish a membrane between himself and his patient, and in the patient between the conscious and the unconscious. Otherwise, as Bion initially observed, verbal language might perceived only as an explusive action, so that the analyst's interpretation would be received by the patient as an act without deeper meaning.

Using the notion of projective identification, Bion developed the idea of a conflictual meeting between the “contained” and the “container.” There are “beta elements,” which are *a priori* incapable of assimilation by the infant mind. They are primitive elements, each “a thing-in-itself,” which must be evacuated. The capacity of the container to contain these elements enables them to become mentally experienced by the infant. This “learning from experience” (Bion, 1962) is represented by the model of the mother-baby dyad: the beta elements coming from the baby must be detoxified by the mother, by her alpha function, her capacity for reverie, so that the baby can eventually benefit from its initially inaccessible emotional experience. If the mother is able to contain the infant in this way, then it will be able to make use of alpha elements in order to dream its fears. If the process fails, a “nameless dread” results. After such containment there is emergence of the mental apparatus with its systemic internal distinction between conscious and unconscious and its border with external reality. There is an establishment of a double limit: conscious/unconscious, internal/external.

We can extract the idea of a psychic envelope from this model. It can take two forms. In the context of a healthy mother-infant dyad, the envelope is made up of alpha elements like the “contact barrier,” following the positive effects of the alpha function on the creation of links due to the relationship between the container and the contained. This delineates the conscious from the unconscious; the subject can dream or partake in autoerotic activity. On the other hand, the failure of this process leads to a confused state in which the envelope corresponds instead to a “screen of beta elements,” which is destructive for the subject and his or her relationships.

Marty (2010) holds that psychosomatic pathology involves a failure to contain drive excitations. Mentalization (Marty, 2010; Smadja and Whitford, 2011) is the process of mental transformation enabling one to manage these excitations. Here the notion of a “protective shield” provides us with a basis for understanding the disturbance of the “biological logic” of somatic functioning. The establishment of the preconscious entails a new organization of the psyche. In Freud, the preconscious is the part of mental apparatus where there is a meeting of unconscious representations of objects and feelings with conscious representations of words. The absence of a protective shield results in a difficulty in linking words with feelings, and in “operative thinking” (“*pensée opératoire*”). Psychosomatic disorders are therefore seen as consequences of the overwhelming of the protective shield, with a consequent lack of differentiation between the body and the mind.

This model of the mother as the protective shield also provides an approach to the psychopathology of the infant. Kreisler (1977) classified etiologic influences from a psychosomatic view into two orientations. One is due to an overload of excitations that penetrate the protective shield, the other to the deficiency or absence of the shield, which generates more severe functional disorders and primary depression. Both are sources of psychosomatic disorder. The body becomes disorganized when excitations cannot become mentally experienced. This is particularly clearly observed in infants (Debray et al., 2005). With this in mind, Debray (1998) considers the psychosomatic balance of the father-mother-baby triad and stresses the need to protect the narcissism of the parents, which provides the basis upon which the protective shield is built.

The concept of the protective shield can also be linked to the balance between sexuality and self-preservation. According to Freud (1950/1905), the mother is the “first seductress.” Sexuality is involved in giving care and in the sharing of pleasures with the baby, both of which invest the mother's libido in the baby and induce pleasure in its erogenic zones. Laplanche (1987) stresses the significant role that “maternal seduction” plays in the “infant's anthropological situation.” He regards this as a universal situation that explains the emergence of the infant's libido.

This maternal seduction is an enigma for the infant. Indeed, the mother's affection partly represents the vestiges of her own history and adult sexuality, as marked by the repression of her own infantile sexuality. This enigmatic maternal seduction will therefore fuel the infant's epistemophilic drive, its curiosity and desire to know, and so the development of its further sexuality and ways of thinking. The other effect of this shared pleasure between mother and baby is the baby's ability to develop its own auto-eroticism; it can now reproduce this pleasure for itself. The infant's libidinal activity thereby produces an erogenic body, which constitutes primary narcissism. However, for both these developments to take place healthily an adequate protective shield is required. If seduction is a necessity for the baby, not enough or too much can cause traumatic confusions between self-preservation and libidinal satisfactions (Gutton, 1997).

## The psychic envelope and the limits of the mental apparatus

We have seen that the idea of a “psychic envelope” can be found in the role Freud assigns to a “protective shield,” and that this kind of conceptualization is continued, albeit in differing ways, in the work of Winnicott, Bion, Marty, and Laplanche. Whichever discussion one chooses, in the absence of such a shield, trauma is liable to cause confusion in the various limits that constitute the mental apparatus: within/without, inner/external, conscious/unconscious, body/mind or self-preservation/libidinal satisfaction. The multiple dimensions of these limits suggest the real complexity of the problems to which the concept of a psychic envelope is addressed. We will consider some aspects of this further via the notions of Skin-Ego, psychic skin and narrative envelope.

## The skin-ego (anzieu): the first outline of the notion of an envelope

Anzieu found that some patients could not use the traditional framework of psychoanalysis, in which successful analysis depended upon the patients' ability to use verbal language in free association in such a way as to represent their own traumas. He initially develops a “transitional psychoanalysis” employing a “prosthesis-framework” for these cases. As he says

What the patient no longer tolerates in the usual analytical framework reveals the early impediment of the environment which has left its mark on his Self. In this case, a new framework must be created by the two contracting parties (the psychoanalyst and the patient), an intermediary between the traditional psychoanalytical framework, which remains the objective of the psychoanalyst, and the prosthesis-framework, precisely adapted in order to compensate the shortcomings of the patient, who claims it explicitly or implicitly (Anzieu, 1979, p. 203, author's translation).

The psychoanalyst taking Winnicott's perspective must initially be able to listen and to take into account the “needs of the Ego.” The traditional framework, based as it us on the patient's ability to use language on the couch, does not enable this. For example lying on the couch without seeing the analyst might exacerbate the patient's primitive anxieties, particularly his paranoid fears, rendering language as used in this position an insecure medium.

Thus Anzieu recommended adjusting the framework when he developed the notions of the Skin-Ego and the psychic envelope. For instance, the transformation of the setting to a “face to face situation” may make the patient more confident. The patient can then experience recognition in the psychoanalyst's face, and can read there the relations between words and affects. All the manifestations of the body can then be assigned meaning, and, of course, the maternal transferences are more acute. Interpretations were also adjusted to the patient's level of symbolization. When the patient cannot put early traumas into words the psychoanalyst may follow the recommendation of Freud (1950/1937) and offer a construction that helps him to imagine the situation. The “group situation” (Kaës, 2007) is another adjustment of the framework.

Anzieu's idea was to rework the standard cure to cater for the existence of a more archaic, more corporeal Ego. The primary differentiation of the ego was seen to emerge from the senses of the skin, which in turn would take on new meaning at the psychic level, inside a symbolic system. In this Anzieu opposed the idea that Bowlby could be said to have introduced a new drive that would precede the oral libido; on the contrary, he considered the two dimensions to be complementary: “in my opinion, the relation between oral drive and the skin constitutes the prototype of all the figure-ground relations” (1974a, p. 152, author's translation). It is the weakness of the Skin-Ego that leads the psychoanalyst to adopt a framework that is less reliant on language. It is important to consider this methodological context to understand both the emergence and the interest of the notion of an envelope.

When explaining the Skin-Ego, Anzieu (1989) also relied on knowledge developed when working as a psychologist in a medical ward (the “envelope” is an item of the Rorschach scale). He refers to the work of Brazelton, who describes how the infant needs an envelope after birth to maintain its homeostasis. The core of his conceptualization relies, however, on the work of Winnicott, with the biological foundation of the Skin-Ego being related to the “needs of the ego.” His concept concerns the development of psychological functions that are closely related to the physiological functions of the skin: those of barrier, surface of meaning and filter (Anzieu, 1974b). Some eight or nine further functions were proposed and developed later (Anzieu, 1989, 2011).

If this development is impaired, then the patient's attention becomes focused on what Anzieu (1990) “formal signifiers” as opposed to linguistic signifiers. These are perceptual, sensory or kinetic traces related to traumatic failures in maternal care. (Anzieu gives the example of “a bag gets a hole in it,” “a skin which shrinks,” etc.). In therapy the characteristics of the Skin-Ego are taken into account by identifying these “formal signifiers”^1^.

### Psychic envelopes

The envelope concept follows on naturally from Anzieu's Skin-Ego. The metaphor of the “Mystic Writing-Pad” (Freud, 1950/1925) is used to define the two sheets of the Skin-Ego: an external face which receives inscriptions but does not store them, which resists destruction but remains rigid; a malleable inner face, which stores the inscriptions, the meaning. The two sheets of the Skin-Ego become envelopes. This double aspect of the envelopes, the external face of the inscription and the inner face of the protective shield, makes it possible to consider various psychopathological configurations depending on the failure of the layers.

The “prohibition to touch” (Anzieu, 1989) explains the structuring of very early relationships, before the oedipal prohibition. It indicates how the infant quits the fantasy of a “common skin” to accede to the Skin-Ego. The first forms of care are the most tactile; the switch to verbal representation requires a distancing, a prohibition, which enables the baby to abandon the “body-to-body exchanges” with mother, so that it can speak and becomes individualized. On this basis, the oedipal prohibition will become possible.

Anzieu showed that this prohibition has a double trigger: initially it relates to the skin and to touch. Later, when the toddler has acquired abilities which lead it to actively explore things and its surroundings, it more specifically concerns what the hand can touch. The first form of prohibition helps the infant to abandon the “intra-uterine fantasy” and to reach the “fantasy of a common skin.” Due to the second form of prohibition, the child abandons the “fantasy of common skin” and acquires a Skin-Ego, an inner world that can contain psychic conflicts without the risk of spilling over. It can be considered a “symboligenic castration” (Dolto, 1984): it is a prohibition that produces a representation, a symbolization, instead of an “amputation” of the body image. The infant can have access to a representation of the differentiation between his body and the mother's body, a symbol is created, it is positive to his development. On the other hand, the failure of this process leads to a confused state of the infant's body image, the infant cannot imagine the differentiation, the prohibition is felt as an intrusion, as an attack. As the incest prohibition at the time of oedipal complex, the “prohibition to touch” could have a helpful role at the time of the primary development of the self.

Envelopes are constructed during the earliest maternal care-giving. They are initially sensory like those related to the senses of smell, touch, thermal perception or sight. Following René Spitz (1965), who distinguished the first proximal senses (touch and smell) from the distal senses (sight), Anzieu advanced the idea of the precession of the touch and smell envelopes over the one for sight. The works which he directly inspired are focused on sensory qualities such as vision, sound and voice (from Anzieu et al., 1993; Gimenez, 1994; Lecourt, 2004).

Anzieu puts two analytical rules—the rule of abstinence and the rule of free associations—and two sheets in relation to each other. The abstinence is in relation with the inner side of the protective shield and the rule of free associations with the outside of the inscription. The progressive differentiation of these two sheets allows the neurotic to use the classic framework and its rules (as described before by Winnicott) whereas disturbances in the differentiation or the establishment of these sheets requires an adjustment to the framework to adapt it to the patient's abilities to symbolize.

Anzieu was also a great theoretician of group psychoanalysis. He made a transition from psychosocial approaches to psychoanalytic approaches to the group. The group envelope concept was not initially part of his thinking; he introduced it later and it thus featured only in the second edition of his book on groups (Anzieu, 1981). His theory shows how the limit between the inside and the outside of the group is constituted. Taking a fresh look at Freud's text Freud (1950/1921), he showed that this limit was based on the ideal of the Ego as shared by all those participating in the group. It is not to be confused with a fantasy and Anzieu gives it a special, structural place. In the same way as the Oedipus complex can organize a group around a symbolic law, the group envelope organizes it according to the filters and filtered content between the inside and the outside of the group. It thus designates a “meta-organizer” of the group's imaginary life. This notion is in common use in group theory.

### Conclusion: a topological perspective on early trauma

Anzieu's conceptualization explicates the relationship between the idea of a protective shield and trauma. The theory of a Skin-Ego made it possible to establish the concept of a psychic envelope. The mark of early childhood traumas can be seen in the flaws of the adult's Skin-Ego, in the quality of the adult envelopes. Whereas in Freud the notion of a traumatic overflow was mainly envisaged according to its intensity, Anzieu emphasizes the topographical dimension of trauma. Trauma must be investigated for the Ego to be reconstructed, so that it can recover its capabilities for synthesis, thinking, and using language.

This model, primarily constructed from abnormal adult psychology, is a powerful tool for considering abnormal child and infant psychology. However, Anzieu's infant remains a “baby reconstructed” from adult analysis. This leaves a number of questions unanswered. For example, how do these various types of envelopes fit into place? Is the skin the only “model” of the existence of envelopes? Can we isolate in the infant an envelope centered on a single, visual, tactile or olfactory meaning as Anzieu does? With the infant, do we not rather see the development of an intermodality of all the senses, even earlier than Anzieu suggests?

We will now approach Houzel's conceptualization, which introduces a notion of an envelope that can be regarded as detached from the metaphor of the skin. After this we will consider Stern's re-conceptualization, with the aim of establishing the envelope as a narrative.

## The “psychic skin” (bick) and the conceptualization of the infant envelope (houzel)

Houzel can be credited with truly establishing the concept of the “psychic envelope” (1987, 2005, 2012). He connected it with the early contributions of Bion, Bick and post-Kleinian psychoanalysts, so enabling it to be related more directly to the infant's body image.

Following the work of Klein on the internal world of the baby, Bick highlighted the importance of the first “psychic skin.” She showed the importance of the containing function of the mother for the earliest creation of the baby's self (1968, 1986). What we are calling the “envelope” results from the introjection of the mother's containing functions.

“The thesis is that in its most primitive form, the parts of the personality are felt to have no binding force among themselves and must therefore be held together in a way that is experienced by them passively, by the skin functioning as a boundary. But this internal function of containing the parts of the self is dependent initially on the introjection of an external object, experienced as capable of fulfilling this function. Later, identification with this function of the object supersedes the unintegrated state and gives rise to the fantasy of internal and external spaces. Only then the stage is set for the operation of primal splitting and idealization of self and object as described by Melanie Klein. Until the containing functions have been introjected, the concept of a space within the self cannot arise. Introjection, i.e., construction of an object in an internal space is therefore impaired. In its absence, the function of projective identification will necessarily continue unabated and all the confusions of identity attending it will be manifest.” (1968, in Briggs, 2002, pp. 55–56).

This process relates to the very first weeks of the newborn baby's life. When the infant is overwhelmed by primitive anxieties, its psychic space is threatened. Bick (1968) introduced the idea of the “second skin,” which we can call a “defensive envelope.” This defense allows the baby to fight against the fear of not being contained, the terror of a never- ending fall. This is all about a kind of “self-containment.” The Ego defends itself against the fear of “non-integration” which generates catastrophic anxieties, by a “pseudo independence” vis-à-vis the object.

This “psychic skin” is obviously connected to Anzieu's Skin-Ego. In fact, in his writings Anzieu himself quoted Bick. There is however a difference of perspective between the two. Bick was working with children and her concept fits infancy better than Anzieu's, which came from working with adults. The metaphor of the skin is not used in the same way either. Bick tried to show how to understand the development of the mental space in infancy. Those who followed in her footsteps, Meltzer, Tustin and Haag, went on to explain connections between this mental space and the body.

### The construction of the mental space

Meltzer (2004) introduced the idea of “a geography of fantasy,” a mental space that is built in infancy through the encounter with the other and in relation to one's own body. The psychic world can be internal (the self) or external (the others), the body can be experienced as having an interior and an exterior. This space has several dimensions. The development of these different dimensions depends on the ability of the subject to think, love and improve experiences through contact with others. In line with the work of Klein, Meltzer particularly studied the negative effects of intrusive projective identification, especially on internal objects. According to Bion, when the object is an inadequate container, the baby risks pathological identifications concerning parts of his own body.

In line with Bick and Tustin, Haag (Haag, 1990, 2004, 2006; Dubinsky and Dubinsky, 2005) examined this “architecture” by showing the importance of the first folds in the skin of baby's body; at the bodily level, these folds duplicate the ties that the baby has with its mother. Thus, for example, the unification of the split around the sagittal axis between the two halves of the body, the right side and the left side, takes up the “container-contained relationship” which the baby has built with its mother. By establishing the junction between its two hands, the right one and the left one, the baby “carries” its left side by its right side, in the same manner that its mother had carried her baby.

This “geography” of body image is thus highly dependent on the introjection of the mother- baby relationship or, more precisely, on the boundaries which the infant determines for its psychic environment. Haag (1993) specifically studied the importance of the interpenetration of gazes and the function of a “background object.” In eye to eye communication with its mother, the baby's eyes plunge into the depths of her eyes. In this movement of projections “in the head” of the other, the baby encounters a bottom, a capacity, an enigma, a mindful and attentive presence. A very small variation in emotion is returned compared to what was projected. This “returning loop” is at the origin of the feeling of being. The presence of a thinking mother limits the amount of projected emotional variation, and this enables the closing of a “returning loop,” and thus the possibility of the baby's linking its own projections to the returning perception.

The enclosed space or background to the encounter constitutes a sort of primitive mirror for the baby. Without this experience, the baby is at risk of losing its limits and of resorting to hallucinations, stimuli from hard objects, or the like, in an attempt to suppress the anxiety of disappearance, much in the same way as is the case in autism. The “returning loop” represents a primitive form of the back and forth-movement from the core of the self. Since this experience is continually repeated, the first psychic container also has a rhythmic structure. It the “hearth” from which the baby can perceive the external world as different to the internal one. Haag linked these “returning loops” to Stern's narrative envelope, as we will see later.

### The infant envelope as a stabilizer

Houzel took Anzieu's work as a starting point, but added approaches of Bick, Meltzer and Tustin from England and that of Haag from France. He defines the psychic envelope as: “the demarcation line between the internal world and the external world, between the internal mental world and the mental world of others” (1987, p. 24, author's translation). He emphasizes the dynamic aspect of this concept: “The psychic envelope should not be conceived in a static way, but rather as a dynamic system, that allows for a synthesis of dynamic and topographic points of view, i.e., the synthesis of the concepts of force and shape.” (1987, p. 40, author's translation). He uses the concept of “attractor” from catastrophe theory to show this dynamic aspect:

The concept of attractor allows Bion's and Bick's descriptions to be better understood, making the nipple/breast the container of the primitive oral impulses. The nipple/breast does not contain in the sense of a recipient, but allows a stable shape to be provided and therefore a meaning, to the baby's oral impulses; it is an attractor for the dynamic system of these impulses, in this sense, it contains them (1987, p. 41, author's translation).

Houzel extended this account to the level of the family group. The family envelope is a…

…group structure common to the members of the same family, which ensures the succession of generations and their differentiation, which allows for the complementarity of maternal and paternal parental roles, which guarantees the constitution of the basic identity and of the sexual identity of each of the children, and which finally contains in respect to the same filiation all the members of the family and leads them to share the same feeling of belonging (2005, p. 136, author's translation).

In this perspective the psychic envelope is thought of as integrating the mental work of both maternal and paternal containment. The difference between the generations and between the sexes allows for the stabilization of the family envelope. In particular, life threatening issues of transgenerational repetitions in mothers with babies suffering from deprivation, which Selma Fraiberg et al. (1975) described as “ghosts in the nursery,” are to be contained and transformed by the family envelope. When families encounter difficulties, in cases of deprivation, negligence or placement of children in care, for example, professionals must work together to reconstruct the stability of the envelope so as to guarantee and protect the identity of all the members of the family and especially that of the suffering children. Houzel thus introduced the idea of an “extended envelope” (2005, p. 140) when the various professionals around the family work together to help the family to be less subject to the influence of massive projective mechanisms and transgenerational pathologic repetition.

### Conclusion: the dynamic containing function of psychic envelopes

Houzel worked toward a second definition of psychic envelopes, which takes into account the mental life of infants. After the topographical perspective developed by Anzieu and the emphasis on narcissism, he insists on the dynamic and drive point of view in the construction of envelopes. Pathology related to envelopes derives from failures in the containing function of the mother, the parents, the family at large and/or of health workers.

Observational studies of both infancy and autism illustrate how the envelope concept can be related to the development of the infant's body image. Haag describes this development in its various stages and processes. It may also be of interest here to cite the work of Dolto (1984) on body image. Even though she does not explicitly refer to an envelope, she clinically describes processes similar to those noted by Haag, though using a conceptual framework derived from Lacan. Likewise studies of parental interactions and projections into infants show possible paths for exploration of this containing function.

The singular fantasies of the baby result from a dynamics that is due to the containing role of the mother. The behavioral interactions of mother and baby carry the imprint of fantasy and unconscious mental life (Kreisler and Cramer, 1981; Lebovici and Stoleru, 2003). The baby receives a “family mandate” (Lebovici, 1998), which can confirm expectations or repair dramas from previous generations. The shade cast by the mother reflects the “violence of her interpretation” (Aulagnier, 2011). The tiniest detail in interaction can become the object of fear, of unconscious inductions, and can indicate the fantasies of the mother. The birth of a child initially upsets the reference of identification of each family member. Parental projections are more or less constraining, with more or less weight being exerted on the baby. Manzano et al. (1999) and Palacio-Espasa (2007) described the various “narcissistic scenarios” of parenting according to the rigidity of the parents' projections into the baby. The baby identifies itself with the place that the parent gives it and that place is more or less constraining. The conception of the infant's psychic envelope can be used to investigate how the infant subjectivises such experiences.

## Narrative envelopes and the “schema-of-being-with” (stern)

Stern (1993, 1995) introduced the idea of a “pre-narrative envelope” to describe the infant's interactions with its mother. The infant's experience was seen to result from a network of schemas of “being-with-another,” including narrative envelopes, scripts, and sensorimotor, perceptual or conceptual schemas. By a process of “re-figuration,” these schemas would give meaning to representations experienced as fantasies, memories or autobiographical representations (1997, p. 132).

Stern especially emphasized the various elements that constitute the experiences of the infant, in particular the affects and what he called “temporary feeling shapes.” This notion refers to a semantic unit. For example, when the infant acts on motives (drinking when thirsty, receiving and adapting to bad news), this is followed by changes in pleasure, emotion and the level of motivation or satisfaction. These changes occur over time with an emergent feeling, which is new, subjectively experienced, singular and complex. This framework of feeling makes it possible to generate the contour and structure of the baby's experience. All of these processes would be at the base of the “internal working model” that constitutes the internalization of the process of attachment.

Here there is an evolution from the nonverbal time of emotional intrigue to the time of verbal representations. The idea of “temporary shape” gives way to “present moments” (Stern, 2004) which are very short, from 1 to 10 s. When musical notes become a tune after the 3rd or 4th note, there is a retroactive effect of unification of what has already occurred (“the past of the present”), and paradoxically this makes it possible to make a leap, to imagine what will happen, by passing on to “the horizon of the future.” According to Ricoeur (1992) psychic time is based on intrigue, the notion of intrigue organizes separate events into an immediately deferred action. This gives shape to feeling; it produces a line of tension that links the schema of affect to those of narrative. Stern regarded time as a kind of sixth sense, something transmodal that adds a certain form of sensuality to the other senses. This conceptualization of infant psychic time can be compared with other works on infant rhythms. Indeed, it could underline the specificity of a very early process of transformation.

These works also led to a model of therapeutic change in which narrative envelopes have a “language” function. *The Boston Change Process Study Group Model* (Stern et al., 2001; Bruschweiler-Stern et al., 2002) investigated therapeutic change within implicit or procedural memory. This was in contrast to traditional interpretative change, which was made possible by the emergence of declarative or autobiographical memory with verbal language. The envelope, as a narrative, can be seen as a kind of primary language.

### Steps in development and communication as mediated by different forms of envelope

Communication successively takes on different forms, related to the development of the self. Communication becomes more and more complex at each step, and also becomes more precise and more abstract. There is an enormous amount of literature on this subject. Different types of studies illustrate the various stages traversed by, for example, the sense of self (Stern, 1985), by intersubjectivity (Trevarthen and Aitken, 2001), by the conscience (Weinberg and Tronick, 1994; Rochat, 2003) and by emotional and social development (Greenspan et al., 2001). These studies could, in our view, provide components for the emergence of infant envelopes. If we consider that, according to Houzel, there is a stabilization of the envelope between the baby and its mother, then we can see each stage of development as a sort of stabilization, due to the maturation of the baby.

The work of Stern (1985) gives us a matrix for this development. He shows how the self becomes more and more complex while giving the baby new vehicles of communication with others. The baby feels its authentic self at the same time that it perceives the potentiality of an outside, of another mind. Being attentive to oneself presupposes being able to perceive outside oneself. Intrinsically, the self is dependent on the quality of caregivers and it is built inside the relationship that the baby develops with its mother.

While defining these meanings for the self very precisely, Stern did not seek to establish correspondences between different communicative methods of the infant and different stages or modes in the envelopes. It is possible to identify different stages that constitute various modes of being in communication with oneself and others (Mellier, 2012). Psychic envelopes may be distinguished according to these various types of operations that involve intersubjectivity, maturation and the corporeity of the baby. Various forms of envelope could be distinguished through the baby's communicative modes. The stabilization of its mode of communication seems due to the fact that the baby shares and associates in a privileged, but non- exclusive, way, particular components of its experiences: sensations, emotions, states of mind, actions, words and then language. Step by step the baby learns how to share and to associate its experiences until it reaches the point of abstraction at which it begins to talk. In this process its Skin-Ego is created little by little, by successive traces, by the unfolding of its experiences. So the development of the sense of self (Stern, 1985) guides us toward four possible forms of envelopes in infancy:

- Envelopes with a sensory basis. Stern describes the “emergent self” in the field of the emergent interpersonal bond; initially the infant has to cope with difficult experiences, with a world of perceptions, of sensations, which it must decipher. Trevarthen (2001) underlines the innate intersubjectivity of new-born, what we call “primary intersubjectivity.” Rochat (2003) introduces the idea of an “ecological self”: the infant is feeling its body as an entity different from its environment. This stage is deeply connected with the construction of the “psychic skin” established by Bick (Mellier, 2010). The breast is the first attractor between the mother and her baby (Houzel, 2005). Perceptions are constructed at the same time in the self and the psychic space. For Anzieu (1989) this is the first step in forming the Ego-skin on the basis of sensory perception (touch, smell, vision). For the infant this is the first differentiation from the mother's psychic space.
- Envelopes with an emotional basis. According to Stern, after the “core self” (at about 2 or 3 months) we enter the field of the interpersonal bond: the baby enters the “immediate social world” with its mother (at about 7–9 months) by learning in a quasi-choreographic way it part in the subtle movements between it and its mother. Trevarthen (2001) introduces the concept of “proto-conversation” which will become on what Stern calls “affect attunements.” This is the beginning of “narrative envelopes,” and is connected with the “fantasy of common skin” described by Anzieu, and the emergence of Kleinian paranoid-schizoid and depressive positions. Spitz's second organizer, the fear of the stranger, is always a pertinent point. For the infant this is the second differentiation from the mother and the parental psychic space.
- Envelopes based on “state of mind.” According to Stern, this is the “subjective self” within the intersubjective interpersonal field (secondary intersubjectivity): the baby discovers that it has a mind and that others also have a mind (Theory of Mind). This is a great step in development, according to Bowlby (1988) that of the internalization of attachment figures. It is at this stage that we can imagine the beginning of the second form of the “prohibition to touch” introduced by Anzieu. For the infant this is the third differentiation from the mother and the parental psychic space.
- Envelopes based on actions that produce representations. According to Stern, this is the “verbal self” (at about 15 months) within the field of the verbal interpersonal bond. With words, the baby discovers that sound symbols open up new prospects to the imagination and communication while bringing into question its former non-verbal worlds. Trevarthen's (2001) work is interesting because he stressed the importance of the companion in infancy and of cooperation. We can also recognize the third organizer, the utterance of “no,” as stressed by Spitz, and the complexity of the passage to verbal language (Vivona, 2009). Later, the child takes a major step into the “world of stories” (Stern, 1990): the child is able to imagine its own story in relation to what happens to others. For the infant, this is the fourth differentiation from the mother and the psychic space of the closed family.

### Conclusion: the question of genesis of envelopes in infancy

The hypothesis that different forms of envelope mediate different stages of development and communication requires further work. Many questions remain unanswered, for instance how can we explain the change from one mode of communication to another, from one envelope to another? Is it merely the result of biological maturation? How do aggressive impulses affect the process of differentiation? Is development independent of relations within the parental dyad? Is it possible to talk of development in infant envelopes without making development a central component?

This last question raises others. According to Green (Sandler et al., 2000), empirically-based developmental research is in danger of destroying the specificity of psychoanalysis and risks leading to oversimplification. Again, psychoanalysis and neurosciences are two different fields (Bazan, 2011), two different ways of explaining the same phenomena, but real questions remain concerning the importation of a concept from one field to the other. The successful conceptualization of psychic envelopes in the field of psychoanalysis depends on one major question: how can we envisage the earliest form of an infant's unconscious world? There is no space here to deal with this thorny problem, but have no doubt that it cannot be ignored (Roussillon, 2011). Keeping psychic envelopes within the analytical framework depends on identifying the psychic conflicts at work for the infant in its relations as well as the processes by which they are elaborated.

## Toward a program of elucidating the role of psychic envelopes

As we conclude this review of the literature, we hope we have shown that the psychic envelopes of an infant can be helpfully defined along the lines of economic, topographical, dynamic and genetic perspectives. The point of departure for our review was the Freudian idea of a “protective shield,” which has undergone the series of developments we have charted via the concept of a psychic envelope. Throughout our review, we have illustrated and discussed the concept of psychic envelopes in the infant and posited the primary economic, topological, dynamic and genetic issues pertaining to this concept. The question of different types of envelopes remains open to discussion as does the question of their transformation, especially in pathological cases. This concept could be further elucidated through work on infant body image and the links with its parents and caregivers. We hope, through this review of the literature, to have laid the foundations for future research in this crucially important field.

### Conflict of interest statement

The author declares that the research was conducted in the absence of any commercial or financial relationships that could be construed as a potential conflict of interest.

## Footnotes

^1^This conceptualization is related to the problem of language and of interpretation. Compare Rosolato's “demarcation signifiers” (Rosolato, 1992) or Aulagnier's “pictograms” (2011).
